# A modeling approach to evaluate the balance between bioactivation and detoxification of MeIQx in human hepatocytes

**DOI:** 10.7717/peerj.3703

**Published:** 2017-09-01

**Authors:** Victorien Delannée, Sophie Langouët, Nathalie Théret, Anne Siegel

**Affiliations:** 1 UMR 6074 IRISA, CNRS, INRIA, University of Rennes 1, Rennes, France; 2 UMR Inserm U1085 IRSET, University of Rennes 1, Rennes, France

**Keywords:** Mathematical modeling, MeIQx bioactivation, Xenobiotic metabolism, Systems Toxicology

## Abstract

**Background:**

Heterocyclic aromatic amines (HAA) are environmental and food contaminants that are potentially carcinogenic for humans. 2-Amino-3,8-dimethylimidazo[4,5-*f*]quinoxaline (MeIQx) is one of the most abundant HAA formed in cooked meat. MeIQx is metabolized by cytochrome P450 1A2 in the human liver into detoxificated and bioactivated products. Once bioactivated, MeIQx metabolites can lead to DNA adduct formation responsible for further genome instability.

**Methods:**

Using a computational approach, we developed a numerical model for MeIQx metabolism in the liver that predicts the MeIQx biotransformation into detoxification or bioactivation pathways according to the concentration of MeIQx.

**Results:**

Our results demonstrate that (1) the detoxification pathway predominates, (2) the ratio between detoxification and bioactivation pathways is not linear and shows a maximum at 10 µM of MeIQx in hepatocyte cell models, and (3) CYP1A2 is a key enzyme in the system that regulates the balance between bioactivation and detoxification. Our analysis suggests that such a ratio could be considered as an indicator of MeIQx genotoxicity at a low concentration of MeIQx.

**Conclusions:**

Our model permits the investigation of the balance between bioactivation (i.e., DNA adduct formation pathway through the prediction of potential genotoxic compounds) and detoxification of MeIQx in order to predict the behaviour of this environmental contaminant in the human liver. It highlights the importance of complex regulations of enzyme competitions that should be taken into account in any further multi-organ models.

## Introduction

Heterocyclic aromatic amines (HAA) are environmental and food contaminants that are formed during cooking of meat and fish, burning of tobacco and diesel exhaust (International Agency for Research on Cancer, 1987). Based on toxicology studies, they have been classified as probable or possible human carcinogens (Group 2A and 2B) by the International Agency of Research on Cancer. Among the HAA, 2-Amino-3,8-dimethylimidazo[4,5-*f*]quinoxaline (MeIQx) is one of the most abundant found in cooked foods, with the level of human exposure estimated to be as high as 2.6 µg of MeIQx per person per day (Ni et al., 2008).

**Figure 1:**
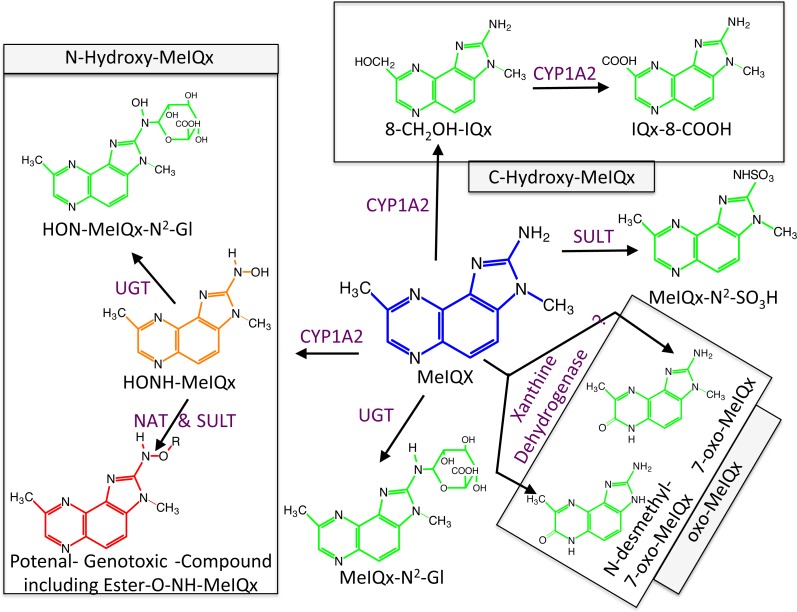
Global MeIQx Metabolism pathway (Langouët et al., 2001). The metabolism of MeIQx is composed of metabolites involved in the detoxification pathways (printed in green) and components of the bioactivation pathway (printed in orange and red). Full chemical names: MeIQx, 2-amino-3,8-dimethylimidazo[4,5-*f*]quinoxaline; HONH-MeIQx, 2-(hydroxyamino)-3,8-dimethylimidazo[4,5-*f*]quinoxaline; MeIQx-N^2^-SO3H, N^2^-(3,8-dimethylimidazo[4,5-*f*]quinoxalin-2-yl)sulfamic acid; MeIQx-N^2^-Gl, N^2^- (*β*-1-glucosiduronyl)-2-amino-3,8-dimethylimidazo- [4,5-*f*]quinoxaline; 8-CH2OH-IQx, 2-amino-8-(hydroxymethyl)-3-meth-ylimidazo[4,5-*f*]quinoxaline; IQx-8-COOH, 2-amino-3-methyl-imidazo[4,5-*f*]quinoxaline-8-carboxylic acid; HON-MeIQx-N^2^-Gl, N^2^-(*β*-1-glucosiduronyl)-2-(hydroxyamino)-3,8-dimethylimidazo[4,*f*-f]quinoxaline; 7-oxo-MeIQx, 2-amino-3,8-dimethyl-6-hydro-7H-imidazo[4,5-*f*]quinoxalin-7-one; N-desmethyl-7-oxo-MeIQx, 2-amino-6-hydro-8-methyl-7H-imidazo[4,5-*f*]quinoxalin-7-one.

Metabolism of MeIQx has been investigated in human hepatocytes few years ago and the data clearly demonstrated that MeIQx is actively transformed into a number of metabolites, which are either detoxification products or potentially reactive metabolites (Langouët et al., 2001). Our previous data identified for the first time the formation of 2-Amino-3-methylimidazo[4,5-*f*]quinoxaline-8-carboxylic acid (IQx-8-COOH) as a major detoxification pathway catalyzed by two successive CYP1A2 oxidations with 2-amino-8-(hydroxymethyl)-3-methylimidazo[4,5-*f*]quinoxaline (8-CH_2_-OH-IQx) as an intermediate (Fig. 1) (Turesky et al., 2002). This new metabolite was predominant at levels approaching human exposure while it was minor at higher concentrations. As usual, the mutagenic and carcinogenic metabolites are formed through N-oxidation reactions which are catalyzed by CYP1A2 in the human liver (Gu et al., 2010). Further activation of the N-hydroxylamine metabolites (HONH-MeIQx) occurs by esterifications catalyzed by N-acetyltransferases (NAT) and sulfotranferases (SULT) leading to the formation of several potential conjugates identified as Ester-O-NH-MeIQx in Fig. 1. These conjugates are able to form reactive nitrenium ions able to bind DNA at the N2 and C8 position of guanine (Turesky & Le Marchand, 2011). The reactive HONH-MeIQx metabolites can also be conjugated by UGT forming N2 glucurono-conjugates (HON-MeIQx-N^2^-Gl) able to be eliminated (Fig. 1). Detoxification is also described through MeIQx-N^2^-SO_3_H and MeIQx-N^2^-Gl formation (Fig. 1). N-desmethyl-7-oxo-MeIQx and 7-oxo-MeIQx are probably formed by the reaction catalysed by xanthine dehydrogenase and are less toxic than the parent compound. CYP1A2 catalyzes reactions involved in both the metabolic activation and detoxification pathways of this procarcinogen in humans (Fig. 1). The formation of MeIQx DNA adducts in human hepatocytes are consistent (2 adducts per 10^6^ bases) (Nauwelaers et al., 2011).

There is evidence that demonstrates the importance of human biological models to study biotransformation of environmental contaminants (Turesky & Le Marchand, 2011). Most of them are very difficult to handle and limited studies exist on HAA metabolism in humans including MeIQx. Although several tissues might be able to metabolize MeIQX, with for instance possible formation of DNA adducts in colon, rectum and kidney, liver is by far the most metabolically active tissue in the biotransformation of MeIQx experiments were performed in human primary hepatocytes. Moreover, the liver is the primary target organ for MeIQx-induced tumors in the rat (Kato et al., 1988; Kushida et al., 1994; Rich et al., 1992). Therefore, primary human hepatocytes are considered as state of the art cell culture models to study the behaviour of xenobiotic in the human liver, however their availability is very limited (Langouët et al., 2002). This limitation led to the development of predictive approaches in order to reduce experimental studies using human hepatocytes. System toxicology approach is a novative and promising area and numerous topics have been recently documented in a special issue of Chemical Research in Toxicology (Sturla & Hollenberg, 2014). Such integrative and modeling approaches allow predictions of xenobiotic metabolism and enlighten complex biological processes. Indeed, some complex biological mechanisms have been experimentally shown this last years (Masubuchi et al., 1993; Pritchett, Kuester & Sipes, 2002), but to our knowledge the bibliography concerning the study of the consequences of such mechanisms on the bioactivation is limited. At this present time, a lot of studies aimed to understand the regioselective change has been carried out. These studies attempt mainly to explain regioselective process by performing amine acid mutations. Indeed, some residues are important to orient the substrate in the catalytic site and mutation of these residues can affect the regioselectivity (Parikh, Josephy & Guengerich, 1999; Josephy, Bibeau & Evans, 2000). In addition, a study has shown that regioselective change could also provide by a structural change of the substrate affecting the preferred formation of a metabolism product (Upthagrove & Nelson, 2001).

In the present study, we implement a system approach to model and understand the effects of a complex mechanism affecting the balance between bioactivation and detoxification. To deepen this assessment, we focus our study on MeIQx metabolism, which has been demonstrated to modulate its detoxification in function of it concentration by an unknown mechanism and without knowing the effect on bioactivation.

**Figure 2:**
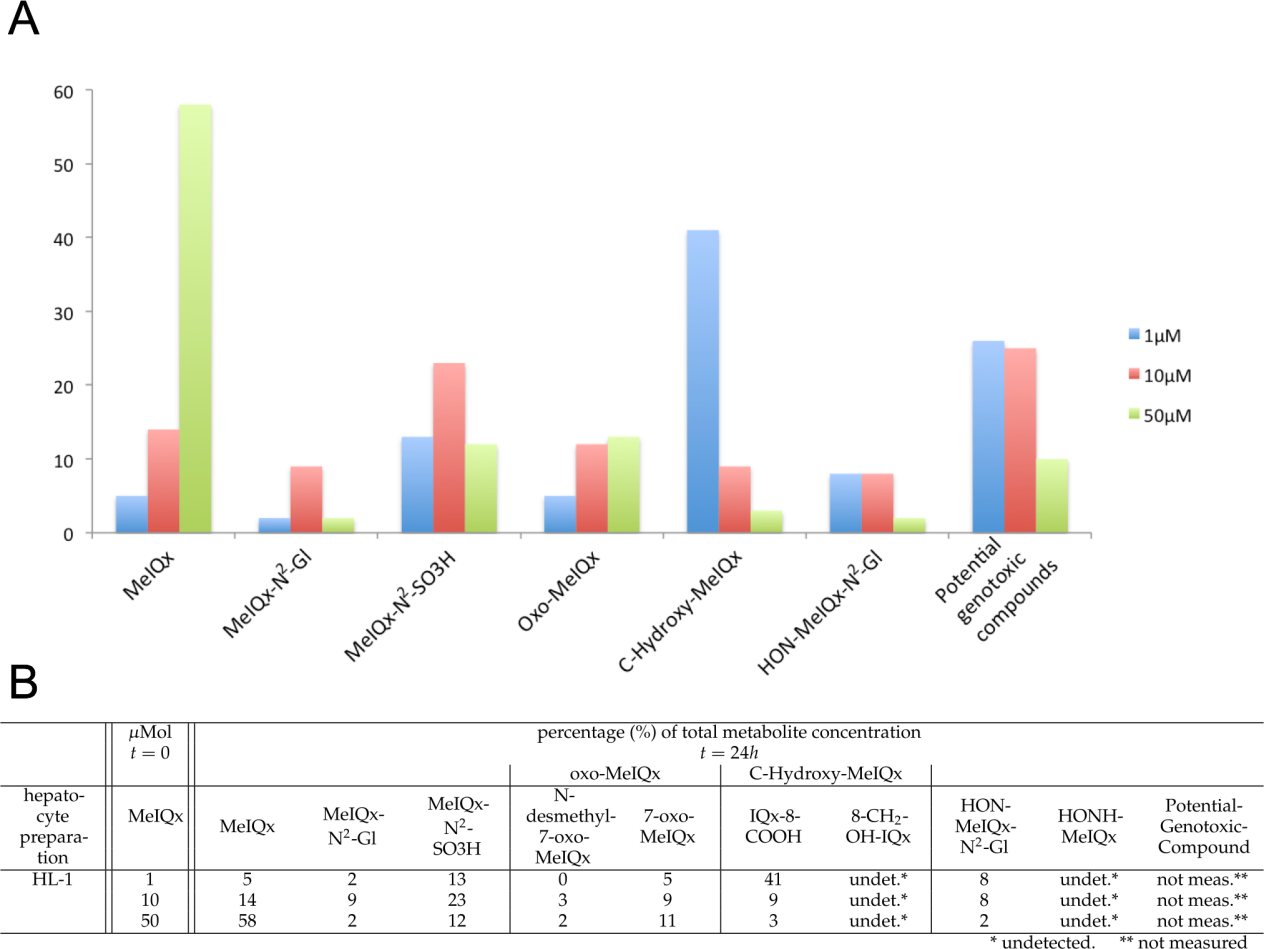
Distribution of MeIQx Metabolites as a function of MeIQx treatment in human hepatocytes (Langouët et al., 2001). Data illustrated by a histogram (A) completed by a table (B) are expressed as percentage of the initial dose of MeIQx.

## Materials & Methods

### Compound concentrations in human hepatocytes in response to MeIQx initial concentrations

The data set used for this present work has been previously published (Langouët et al., 2001). Briefly, human hepatocytes isolated from human liver biopsies were treated with low concentration of MeIQx (1, 10 and 50 µM) not to induce cytotoxic effects (Langouët et al., 2001). The culture media were retrieved at 24 h and the metabolites were purified and quantified by mass spectrometry. Figure 2 shows the percentage of total metabolite quantity for the ten following compounds: MeIQx, MeIQx-N^2^-Gl, MeIQx-N^2^-SO_3_H, N-desmethyl-7-oxo-MeIQx, 7-oxo-MeIQx, IQx-8-COOH, 8-CH_2_-OH-IQx, HON-MeIQx-N^2^-Gl, HONH-MeIQx, and Potential-Genotoxic-Compound. To describe the metabolism of MeIQx with a numerical model based on ordinary differential equations (ODE), we first converted each compound percentage in molar concentration by using a proportional relationship: $[\text{compound}]_{t=24}= \frac{1}{100} [\text{MeIQx}]_{t=0}\ast (\text{percentage of total compound concentration})_{t=24}$.

**Figure 3:**
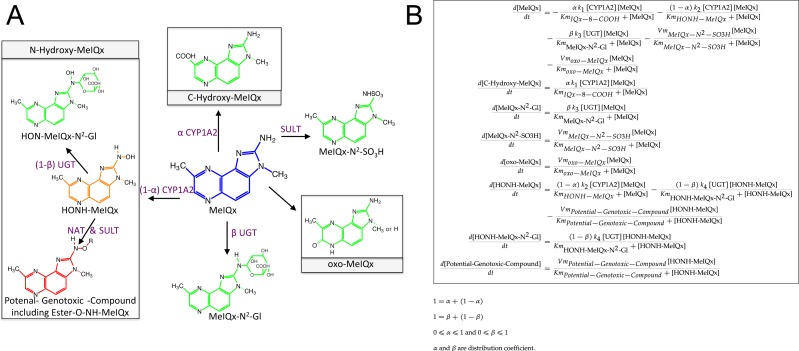
Simplified representation of MeIQx metabolism (A) corresponding to the ODE describing the mathematical model of MeIQx metabolism used (B). The green color corresponds to the detoxification pathway and the red color to the bioactivation pathway as described in Material and Method, Data sample section. Orange represents a very labile metabolite and purple the enzymes catalyzing the reactions.

In order to decrease the final ODE model variability carried out by too large a number of parameters, we operated several reductions based on the following hypotheses:

1. It is assumed that 8-CH_2_-OH-IQx has a transitory effect because it was not detected at *t* = 24 h. Consequently, the pathway that hydroxylates MeIQx into 8-CH_2_-OH-IQx and IQx-8-COOH were merged in one generic reaction which produces a generic compound named “C-Hydroxy-MeIQx”. This generic compound gathers all carbon C8 hydroxylated compounds (Fig. 3).
2. Assuming that the formation of N-desmethyl-7-oxo-MeIQx and 7-oxo-MeIQx is mediated by a same enzyme, probably xanthine dehydrogenase, we introduced a novel generic compound “oxo-MeIQx” which gathers the compounds with an oxo chemical group. This generic compound oxo-MeIQx is produced by a single generic reaction with MeIQx substrate.
3. It is assumed that HONH-MeIQx has a transitory effect because it was not detected at *t* = 24 h.
4. In order to model a closed system, we wish to assume that all MeIQx present at *t* = 0 was either recovered or transformed and distributed between the various metabolites of the system. To that purpose, we introduced a last variable in the system, named “Potential-Genotoxic-Compound”. Its concentration was estimated by summing up all concentrations at *t* = 24 h to the initial dose of MeIQx. Notice, however, that according to the literature, the main non-measured metabolite in the system is Ester-O-NH-MeIQx. Therefore, the variable ”Potential-Genotoxic-Compound” corresponds to the Ester-O-NH-MeIQx and the potential unidentified compounds.

As illustrated in the reduced model (Fig. 3), the concentrations of the new generic compounds were computed as follows.

\begin{eqnarray*}[\text{C-Hydroxy-MeIQx}]& =[\text{IQx-8-COOH}]+[{\text{8-CH}}_{2}\text{-OH-IQx}] \end{eqnarray*}

\begin{eqnarray*}\,[\text{oxo-MeIQx}]& =[\text{7-oxo-MeIQx}]+[\text{N-desmethyl-7-oxo-MeIQx}]. \end{eqnarray*}

Together, we obtained an exhaustive estimation of the concentrations of the different compounds involved in the numerical model at time *t* = 24 h: MeIQx, C-Hydroxy-MeIQx, MeIQx-N^2^-Gl, MeIQx-N^2^-SO3H, oxo-MeIQx, HONH-MeIQx, HON-MeIQx-N^2^-Gl and Potential-Genotoxic-Compound. Quantities for each compounds are depicted in Table 1 except HONH-MeIQx that is assumed to be zero because of its transitory effect.

**Table 1:** Distribution of MeIQx Metabolites formed as a function of MeIQx treatment in human hepatocytes. The metabolite concentrations calculated from the percent distribution and deduced from the known metabolite concentrations at 24 h. Data are expressed as concentration in micromolar.

	*t* = 0	*t* = 24 h								
					oxo-MeIQx	C-Hydroxy-MeIQx				
Hepatocyte sample	MeIQx	MeIQx	MeIQx-N^2^-Gl	MeIQx-N^2^-SO3H	Total	Total	HON-MeIQx-N^2^-Gl	Potential-Genotoxic-Compound	Detoxi fixation	$ \frac{\mathrm{Bioactivation}}{\mathrm{Detoxification}} $
HL-1	1	0.05	0.02	0.13	0.05	0.41	0.08	0.26	0.69	0.377
	10	1.4	0.9	2.3	1.2	0.9	0.8	2.5	6.1	0.410
	50	29	1	6	6.5	1.5	1	5	16	0.313

As a main output of the model, we aimed at comparing the bioactivation pathways with the detoxification pathways according to MeIQx concentration. To that purpose, we introduced the ratio $ \frac{\text{[Bioactivation]}}{\text{[Detoxification]}} $ based on the two variables [Bioactivation] and [Detoxification] computed as follows.

\begin{eqnarray*}[\text{Detoxification}]& =[{\text{MeIQx-N}}^{2}\text{-Gl}]+[{\text{MeIQx-N}}^{2}{\text{-SO}}_{3}\text{H}]+[\text{oxo-MeIQx}]\nonumber\\\displaystyle & +[\text{C-Hydroxy-MeIQx}]+[{\text{HON-MeIQx-N}}^{2}\text{-Gl}] \end{eqnarray*}

\begin{eqnarray*}[\text{Bioactivation}]& =[\text{Potential-Genotoxic-Compound}]. \end{eqnarray*}

### Non parameterized numerical model for MeIQx metabolism

According to the data set and the simplifications depicted in the Method section, we built a ODE numerical model to describe MeIQx metabolism. The model contained eight variables: MeIQx, C-Hydroxy-MeIQx, MeIQx-N^2^-Gl, MeIQx-N^2^-SO3H, oxo-MeIQx, HONH-MeIQx, HON-MeIQx-N^2^-Gl and Potential-Genotoxic-Compound. For the three reactions regulated by an enzyme which was not involved in any other reaction, the concentration of the product was mainly assumed to be produced with a Michaelis-Menten dynamics. Each Michaelis-Menten function was parameterized by a catalytic constant *K*_*m*_ and a maximum rate *V*_*m*_, leading to six parameters: *Vm*_*MeIQx*−*N*^2^−*SO*_3_*H*_, *Vm*_*oxo*−*MeIQx*_, *Vm*_*PotGenotoxC*_, *Km*_*MeIQx*−*N*^2^−*SO*_3_*H*_, *Km*_*oxo*−*MeIQx*_, *Km*_*PotGenotoxC*_.

A main characteristic of the model is that both CYP1A2 and UGT catalyze pairs of reactions leading to competition for the enzyme resources. First, CYP1A2 metabolizes its substrate MeIQx into two products P1 = C-Hydroxy-MeIQx and P2 = HONH-MeIQx. Second, UGT metabolizes both the substrates S1 = MeIQx and S2 = MeIQx-N^2^-Gl into the products P1 = MeIQx-N^2^-Gl and P2 = HON-MeIQx-N^2^-Gl, respectively.

In both cases, an enzyme *E* catalyzes two reactions *R*1 and *R*2. In that context, we introduced two parameters, named *distribution coefficients*, *α* (for the CYP1A2 competition) and *β* (for the UGT competition) to take into account the proportion of total enzyme concentration used in the reactions:

1. *α*[CYP1A2] and (1 − *α*)[CYP1A2] denotes the quantity of CYP1A2 involved in the catalyzing MeIQx into C-Hydroxy-MeIQx and HONH-MeIQx, respectively.
2. *β*[UGT] and (1 − *β*)[UGT]) denotes the quantity of UGT involved in the catalyzing MeIQx into MeIQx-N^2^-Gl and of HONOH-MeIQx into HON-MeIQx-N^2^-Gl, respectively.

**Figure 4:**
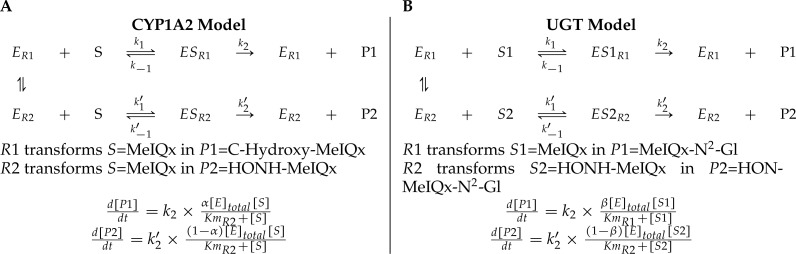
A generic model for the competition of reactions which are catalyzed by the same enzyme. The same substrate MeIQx is transformed into two different compounds by the same enzyme CYP1A2. The symbol *α* denotes the distribution coefficient of enzyme concentration spread in the two reactions (A). The enzyme UGT is involved in the catalysis of two reactions which are in competition with respect to the UGT concentration. This concentration is depicted by the distribution coefficient *β* (B).

Notice that the underlying assumption is that the total enzyme amount of CYP1A2 and UGT is limited. This assumption is supported by former analyses of enzymatic activities estimated for CYP1A and 1B using ethoxyresorufin as a substrate. These experiments evidenced that enzymatic activities showed relatively constant values during the culture (see Table 2 in Langouët et al., 2001). The corresponding generic ODE models are depicted in Fig. 4. In addition to the distribution coefficients *α* and *β*, modeling competitions required to introduce other parameters: four catalytic constants (*k*_1_, *k*_2_, *k*_3_, *k*_4_), the Michealis-Menten constants (*Km*_1_, *Km*_2_, *Km*_3_, *Km*_4_), and the total concentration of CYP1A2 and UGT.

To conclude, the model (Fig. 3) reports the dynamics of eight variables according to 16 fixed parameters (catalytic constants, maximal rates, and CYP1A2 and UGT total concentrations) and two additional parameters named distribution coefficients (*α*, and *β*) which may vary according to the experimental setup.

### Modeling the enzyme competition process according to the initial MeIQx concentration

To evaluate the dynamics of CYP and UGT enzyme distributions between the two metabolic pathways, we implemented different models: a classical Michaelis-Menten model testing the saturation hypothesis and a dynamic model in which the amount of enzyme involved in a pathway can change dynamically in function of the substrate concentration. The main difference between the two hypotheses is related to the dependency with respect to the dose of the coefficients *α* and *β* modelling the enzyme distribution coefficients of CYP1A2 and UGT.

• The *saturation hypothesis* assumes that both the distribution coefficients *α* and *β* are fixed with respect to the concentration of [MeIQx].
• The *dose-dependent hypothesis* assumes that both the distribution coefficients *α*, *β* depend on the concentration of [MeIQx] at any time point, that is, *α* = *φ*(*a*_1_, *b*_1_, *θ*_1_, *n*_1_, [MeIQx]) and *β* = *φ*(*a*_2_, *b*_2_, *θ*_2_, *n*_2_, [MeIQx]). To model these relations of dependency, we used as generic sigmoid function $\varphi (a,b,\theta ,n,S)=a \frac{{\theta }^{n}}{{\theta }^{n}+{S}^{n}} +b$, where *a* and *b* (0 ≤ *a*, *b* ≤ 1) are the boundaries of the sigmoids; *n* = 5 describes the slope of the sigmoid; *θ* is the inflection point of the curve.

### Model fitting, parameters estimation, and *a posteriori* filtering

The parameters of all ODEs numerical models were selected such that the model predictions optimally fitted with the data set from the experiment HL-1 (Table 1) at *t* = 24 h.

For this, we denoted by *t*_1_ = 0 and *t*_2_ = 24 h the two time-points introduced in Table 1. We also introduced *d*_1_ = 1 µM, *d*_2_ = 10 µM and *d*_3_ = 50 µM the initial concentrations of MeIQx used in experiments. We also denoted by *C*_1_, …, *C*_8_ the compounds that include 7 compounds from Table 1 and HONH-MeIQx that is not detected. In this setting, we denoted by ${y}_{k,l,i}^{obs}$ the measured concentration of each compound *C*_*k*_ at time *t*_*l*_ in HL-1 hepatocytes in presence of initial MeIQx concentration *d*_*i*_. Together, we obtained 48 experimental data points.

All models describe the variations of the eight variables *C*_1_, …, *C*_8_ according to time. Each model is parameterized by a set of parameters *θ*, including (i) the 16 fixed parameters of the model and (ii) the parameters allowing to specify the values of the distribution parameters *α* and *β* according to the saturation and dose-dependent hypotheses. The prediction concentration of a compound *C*_*k*_ at time *t*_*l*_ in HL-1 hepatocytes in presence of initial MeIQx concentration *d*_*i*_ is denoted by *y*_*k*_(*θ*, *t*_*l*_, *d*_*i*_).

The PottersWheel toolbox for Matlab software (Maiwald & Timmer, 2008) was used to sample the family of all possible parameters set *θ*. The main steps of the method are as follows. First, a random set of parameters is selected. From this set of parameters, a trust-region algorithm is applied iteratively to decrease the following mean-square distance (Raue et al., 2009), where *σ*_*k*,*l*,*i*_’s depict an internal error linked to the parameter estimation process.

\begin{eqnarray*}{\chi }^{2}(\theta )=\sum _{k=1}^{8}\sum _{l=1}^{2}\sum _{i=1}^{3}{ \left( \frac{{y}_{k,l,i}^{obs}-{y}_{k}(\theta ,{t}_{l},{d}_{i})}{{\sigma }_{k,l,i}} \right) }^{2}. \end{eqnarray*}

When the trust-region algorithm has reached a local minimum, the PottersWheel toolbox reports a *p*-value which allows evaluating the goodness of the selected set of parameters. The set of parameters was considered as relevant if the *p*-value was equal to 1 (most stringent case).

The added value of PottersWheel is to iterate this procedure in order to avoid bias introduced by local minima and to explore the full space of set of parameters. To that goal, the former set of parameters identified by the PottersWheel toolbox was perturbed enough to allow the exploration of new regions of the search space. The trust-region algorithm was again applied iteratively to reach another local best-fit set of parameters which is also stored as a relevant solution when its associated *p*-value equals 1. This procedure was iterated 1,000 times for both the saturation and dose-dependency hypothesis models. To improve the performance of the algorithm, data were transposed in a logarithmic scale prior to applying the fitting procedure. All the parameterized values used to run PottersWheel are detailed in Table S2.

Therefore, the training methods enabled the minimization of the distance between the 48 experimental data points ${y}_{k,l,i}^{obs}$ and the parameterized model predictions *y*_*k*_(*θ*, *t*_*l*_, *d*_*i*_) and resulted in a family of sets of parameters *θ*.

In a second step, the family of reported suitable sets of parameters *θ* was filtered in order to eliminate false positive results. This was done by simulating the behavior of the family of parameterized numerical models with the “radau5” solver associated to the default thresholds Absolute Tolerance =1 × 10^−10^ and Relative Tolerance =1 × 10^−6^. Based on this procedure, we retained only the models with the following characteristics: (i) the formation of all variables follows a monotone dynamics, (ii) In order to limit the variability of models on each single data point, we constrained the species that are the most variable with respect to their measurements, e.g., C-Hydroxy-MeIQx and [Detoxification] at initial dose *d*_1_ = 1 µM, as follows:

\begin{eqnarray*} \left\vert \frac{{y}_{\text{C-Hydroxy-MeIQx}}(\theta ,{t}_{2},{d}_{1})-{y}_{\text{C-Hydroxy-MeIQx},2,1}^{obs}}{{y}_{\text{C-Hydroxy-MeIQx},2,1}^{obs}} \right\vert \leq 0.10,\nonumber\\\displaystyle -0.05\leq \frac{[\text{Detoxification}](\theta ,{t}_{2},{d}_{1})-[\text{Detoxification}]_{2,1}^{obs}}{[\text{Detoxification}]_{2,1}^{obs}} \leq 0.2; \end{eqnarray*}

where $[\text{Detoxification}]_{2,1}^{obs}$ denotes the value of detoxification at time *t*_2_ = 24 h for the initial dose *d*_1_.

Finally, the Detoxification pathway, the formation of Potential-Genotoxic-Compound, and consequently the bioactivation of MeIQx were estimated by simulating the final family of parameterized models for MeIQx initial concentrations equal to 0.05, 0.1, 0.5, 1, 2, 5, 10, 15, 30, 50, 75 and 100 µM during 120 h, in order to evaluate the balance between the two pathways.

## Results

### Model-fitting suggests that the dose-dependance hypothesis is more relevant than the saturation hypothesis

As detailed in Material and Methods, in order to study the metabolism of MeIQx in humans, we set up a scheme of numerical models using ordinary differential equation (ODE) based on the data from the experiment using human hepatocytes HL-1 reported in Langouët et al. (2001). Two hypotheses were introduced to model the enzyme competition process according to the initial MeIQx concentration and tested by first searching for the sets of parameters which best explain the data and then testing their relevance with individual data.

**Table 2:** Best fitting scores for the total ODE model described in Fig. 3, according to the saturation and the dose-dependent hypotheses. This table depicts the fitting score of each metabolic compounds according to the parameter estimation procedure detailed in Material and Methods. The *p*-value is a score reported by the PottersWheel toolbox in order to evaluate the goodness of the best-fitting set of parameters by taking into account the number of datapoints and the quality of the inferred sets of parameters. Local and global Chi^2^ depict more precisely the quality of such sets of parameters. A local score is considered to be valid (✓) when it is lower than 1 and when its value is located between the errors bars computed by The PottersWheel toolbox at *t* = 24 h. A global score is valid when all local scores are valid. In other cases, the scores are considered as invalid ✗ and the corresponding hypotheses are rejected.

	µMol	Best Chi^2^ score per 1,000 fit sequence										
	MeIQx	MeIQx	MeIQx-N^2^-Gl	MeIQx-N^2^-SO3-	oxo-MeIQx	C-Hydroxy-MeIQx	HON-MeIQx-N^2^-Gl	HONH-MeIQx	Potential-Genotoxic-Compound	Local Chi^2^ score	Global Chi^2^ score	Best *p*-value
		**Saturation hypothesis 1**. Does the pathway saturation explain the data?										
		The CYP1A2 and UGT distribution coefficients *α* and *β* are considered as global parameters, which										
		are independent of the initial MeIQx concentration and have the same value for the 3 initial doses of MeIQx										
Fixed *α* and *β*	1	✓ (0.01)	✓ (0.82)	✓ (0.00)	✓ (0.00)	✓ (0.00)	✓ (0.01)	✓ (0.00)	✓ (0.01)	✓ (0.854)	✗ 20.9622	✗ 0.893335
	10	✓ (0.01)	✗ (12.98)	✓ (0.00)	✓ (0.00)	✗ (1.88)	✓ (0.23)	✓ (0.00)	✓ (0.39)	✗ (15.50)		
	50	✓ (0.04)	✓ (0.35)	✓ (0.00)	✓ (0.00)	✗ (2.99)	✓ (0.02)	✓ (0.00)	✗ (1.21)	✗ (4.610)		
		**Dose-dependent hypothesis**. Can we model the dynamic										
		with a function directly dependent of the substrate concentration?										
		The fitting experiments are performed by considering that the distribution coefficients										
		*α* and *β* depend on the concentration of MeIQx										
		*α* and *β* dynamically evolve with MeIQx concentration in function of time.										
		These parameters are modeled by sigmoid functions which depend on the MeIQx concentration.										
*α* and *β* dynamical	1	✓ (0.84)	✓ (0.00)	✓ (0.01)	✓ (0.05)	✓ (0.02)	✓ (0.03)	✓ (0.00)	✓ (0.04)	✓ (0.987)	✓ 2.57838	✓ 1
	10	✓ (0.58)	✓ (0.03)	✓ (0.35)	✓ (0.30)	✓ (0.02)	✓ (0.07)	✓ (0.00)	✓ (0.01)	✓ (1.34)		
	50	✓ (0.04)	✓ (0.00)	✓ (0.00)	✓ (0.00)	✓ (0.00)	✓ (0.01)	✓ (0.00)	✓ (0.19)	✗ (0.248)		

As shown in Table 2, the saturation hypothesis (line 1) cannot be explained with the family of parameters identified by the fitting procedure. Indeed, all models reported by PottersWheel toolbox procedure had a p-value lower than 1, which is too low to be considered as relevant. An explanation can be found in the study of local Chi^2^-scores involved in the computation of the p-value shown in Table 2. Indeed, four local Chi^2^ scores are being found outside the deviation area. In particular, if the distribution parameters *α* and *β* are fixed, the local score for MeIQx-N^2^-Gl and C-Hydroxy-MeIQx is too distant from biological data, respectively, to retain the saturation hypothesis.

On the opposite, the sets of parameters found for the dose-dependent hypothesis (line 2) can reproduce and explain the biological data from Hl-1 experiment. The parameter searching procedure resulted in a family of 260 sets of parameters (also called “fit”) which all equivalently best-fit the data and have a *p*-value equal to 1. Their filtration according to biological criteria resulted in a family of 73 sets of parameters which equivalently explained the HL1 data set and are based on the saturation hypothesis. These results suggest that the distribution of CYP1A2 and UGT between their respective pathways dynamically depends on the concentration of MeIQx. Additionally our data demonstrate that modeling such dependency with sigmoid function is appropriate describing the dynamics of biological processes.

In the following, the 73 corresponding numerical models were used to predict MeIQx metabolism activation at various concentrations. This resulted in 73 prediction curves, each corresponding to a set of parameters.

### Model validation based on literature datasets

Among the detoxification metabolites, the major one is the C-Hydroxy-MeIQx derivatives (Langouët et al., 2001). We first explored the behavior of C-Hydroxy-MeIQx and HONH-MeIQx-N^2^-Gl for short time and low concentrations, corresponding to the usual human exposures. For this purpose, we used different values of parameters corresponding to the 73 valid numerical models.

**Figure 5:**
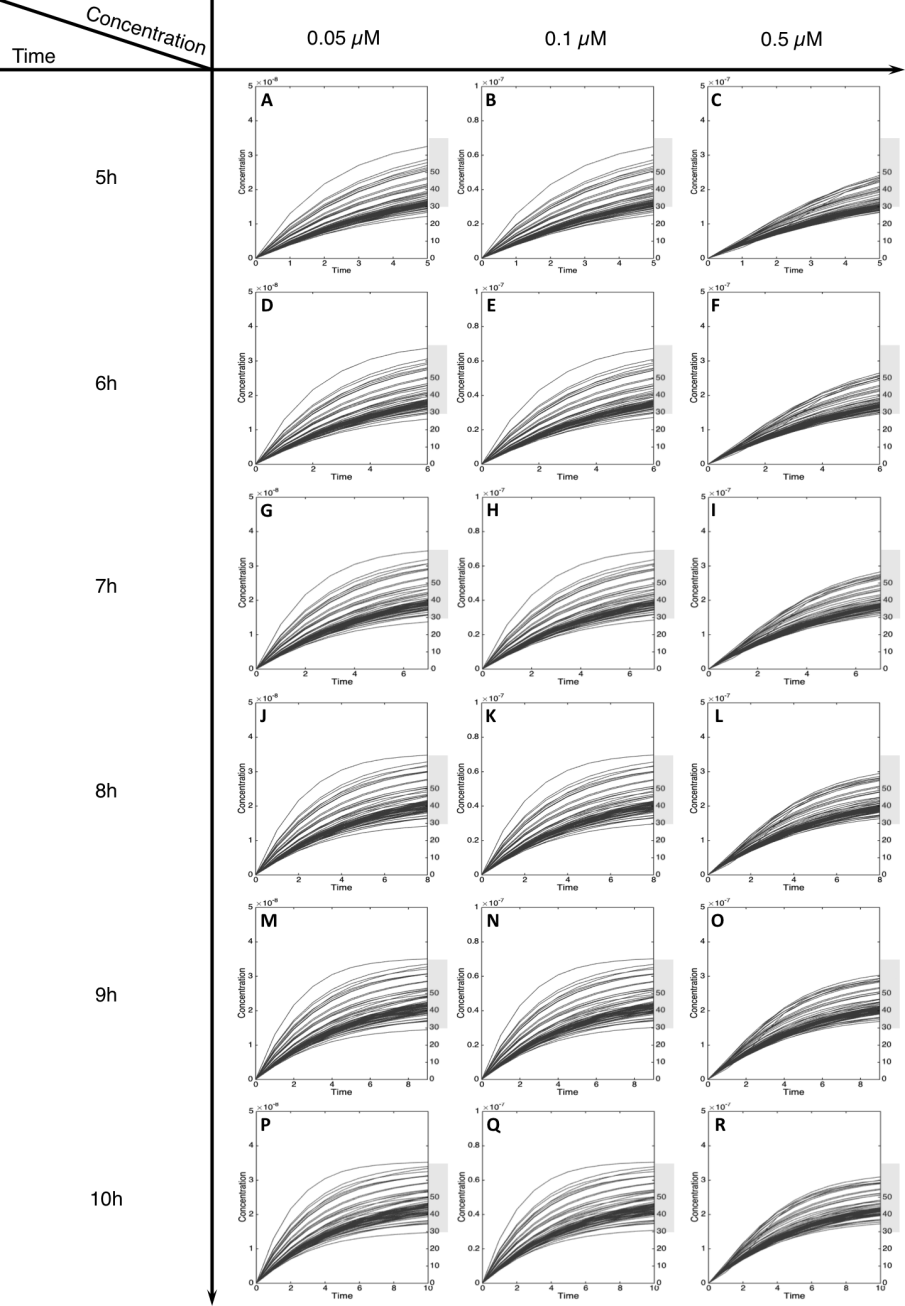
Metabolism of C-Hydroxy-MeIQx at initial concentration of 0.05, 0.1, 0.5 µM after 5, 6, 7, 8, 9, 10 h of exposition. The grey rectangle represents the area between 30 and 70% of the MeIQx metabolized. Concentrations are in Molar on the *y* axis and time in hours on the *x* axis. (A, D, G, J, M, P) C-Hydroxy-MeIQx formation at an initial MeIQx concentration of 0.05 µM (B, E, H, K, N, Q) C-Hydroxy-MeIQx formation at an initial MeIQx concentration of 0.1 µM (C, F, I, L, O, R) C-Hydroxy-MeIQx formation at an initial MeIQx concentration of 0.5 µM.

For MeIQx initial concentrations between 0.05 to 0.5 µM, the formation of C-Hydroxy-MeIQx reaches a plateau (steady-state) before 7 h of treatment (Fig. 5). At this time, for any initial dose less than 0.5 µM, the predicted quantity of C-Hydroxy-MeIQx varies from 30% to 70% of the initial dose. This is consistent with data from Gu et al. (2010) reporting that C-Hydroxy-MeIQx accounted for 35.8% to 73.1% (see Table S1) of the dose of MeIQx measured in urine 10 h after ingestion of 920 ng of MeIQx by human volunteers.

**Figure 6:**
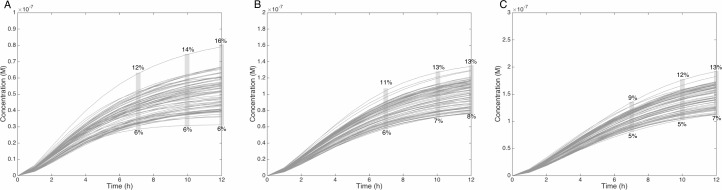
Metabolism of HON-MeIQx-N^2^-Gl at initial concentration of 0.5 (A), 1 (B), 1.5 (C) µM after 7 h of exposition. The grey rectangle represents the range variation of HON-MeIQx-N^2^-Gl in percentage. Stillwell et al. (1999) observed in urine at 12 h that the percentage of MeIQx converted in for a MeIQx dose of 1,500 to 3,000 ng (corresponding to a concentration of 0.7 to 1.4 µM by applying the same proposed method based on equivalence relation for Gu in Table S1) is between 2.2 to 17.1% of the MeIQx ingested dose. By simulating the formation of HON-MeIQx-N^2^-Gl for concentrations of MeIQx equal to 0.5, 1 and 1.5 µM, the maximum obtain range of converted MeIQx into N-OH-MeIQx-N^2^-Glc is between 6 and 12% after 7 h of exposition and 6 and 16% after 12 h of exposition. Concentrations are in Molar on the y axis and time in hours on the *x* axis.

Similarly, when the initial dose of MeIQx varies from 0.5 µM to 1.5 µM, the predicted quantities of HONH-MeIQx-N^2^-Gl reaches a plateau between 7 h and 12 h of treatment. During this time interval, the predicted quantity varies from 6% and 16% of the initial dose. This is consistent with data from (Stillwell et al., 1999) reporting that HONH-MeIQx-N^2^-Gl accounted for 2.2 to 17.1% of the dose of MeIQx measured in urine at 12 h for an exposition between 1,500 and 3,000 ng (that is 0.7 to 1.4 µM) (Fig. 6). These comparisons provide an indirect validation of our modelling approach and highlight the high level of variability in the measured response of the system, which is properly reflected by the choice of a family of ODE models instead of a single one.

### Evaluation of the balance between bioactivation and detoxification

**Figure 7:**
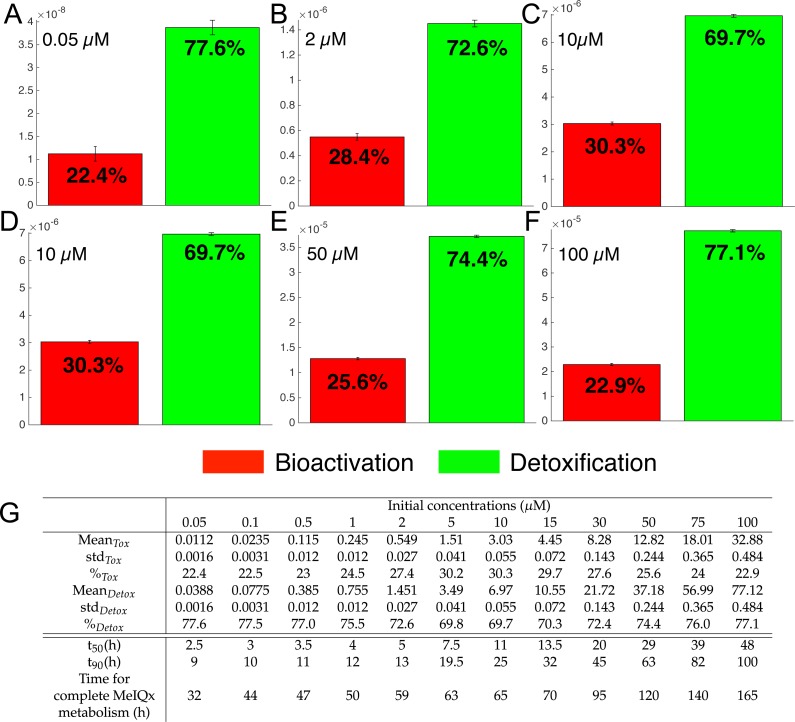
Biotransformation of the MeIQx into bioactivation products (red) and detoxification products (green) with different initial concentrations of MeIQx. A total of 73 models were simulated using 12 initial different concentrations (0.05, 0.1, 0.5, 1, 2, 5, 10, 15, 30, 50, 75, 100 µM) after 180 h (where the steady-state has been reached) of MeIQx exposition. Each histogram illustrates the balance between bioactivation and detoxification products, which gradually favors the bioactivation for MeIQx concentration between 0.05 and 10 µM (A–C) and the detoxification for concentration between 10 and 50 µM (D–F). The importance of each product is expressed in percentage. The table depicts the concentrations in µM of bioactivation and detoxification products for the 73 simulated models (G). The corresponding scores for the 73 models are calculated: average value, standard deviation (std) and corresponding percentage of the MeIQx metabolized into a bioactivation or a detoxification product. In addition, *t*_50_ represents the time where 50% of MeIQx is metabolized and *t*_90_ represents the time where 90% of MeIQx is metabolized. Finally, time for complete MeIQx metabolism is given.

As shown in Fig. 3, the global MeIQx metabolism can be decomposed into detoxificated and bioactivated pathways. The detoxification pathways include C-hydroxy-MeIQx together with all phase II conjugates (green compounds in Fig. 7). In regards, bioactivation corresponds to the formation of Potential-Genotoxic-Compound, that is, Ester-O-NH-MeIQx and the potential unidentified metabolites (red compound in Fig. 7). In order to evaluate MeIQx bioactivation according to its initial concentration, we used our 73 mathematical models to simulate the range of detoxification and bioactivation pathways of MeIQx from 0 to 180 h, when all models have reached a steady-state. For this purpose, we again used different values of parameters corresponding to the 73 valid numerical models.

Figure 7 illustrates the distribution of MeIQx at steady-state into bioactivation and detoxification products expressed either in percentage (Fig. 7A) or in concentrations (Fig. 7B, µM). Our results show that the detoxification pathway always predominates although the balance between bioactivation and detoxification changes in function of MeIQx concentration. More precisely, the percentage of MeIQx transformed into bioactivated pathway increases when the initial concentration of MeIQx switches from 0.05 µM and 10 µM. In contrast, for concentrations greater than 10 µM, the balance decreases progressively when the MeIQx dose increases.

In addition, as depicted in the Table contained in Fig. 7B, we noticed that the duration required to transform 50% of MeIQx corresponds to 10% to 25% of the duration required to reach a complete degradation of MeIQx. For instance, for an initial dose of 0.05 µM of MeIQx, the complete metabolism takes 32 h although only 2.5 h is needed to metabolize 50% of the MeIQx dose and 9 h are needed to metabolize 90% of the MeIQx dose (Fig. 7B).

### Evaluation of the Bioactivation/Detoxification ratio

**Figure 8:**
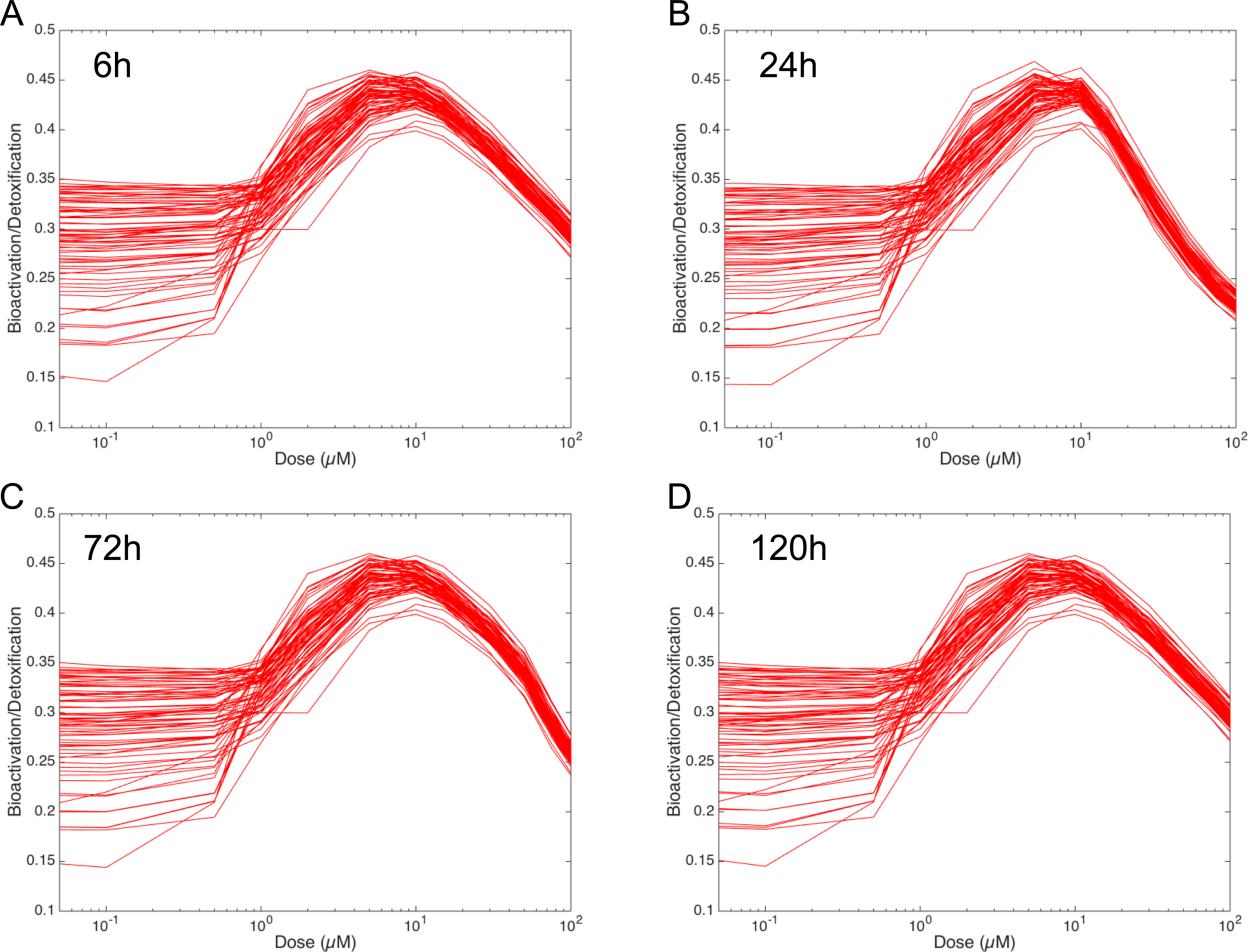
Evolution of Bioactivation/Detoxification ratio as function of concentration of MeIQx exposition. This ratio is illustrated for 73 models simulated using 12 initial different concentrations (0.05, 0.1, 0.5, 1, 2, 5, 10, 15, 30, 50, 75, 100 µM) after 6 h (A), 24 h (B), 72 h (C) and 120 h (D) of MeIQx exposition.

In order to figure out the modulation of the ratio between bioactivation versus detoxification pathways, we plotted the evolution of this ratio between 0.05 and 100 µM at 6 h, 24 h, 72 h and 120 h in Fig. 8. This clearly shows that, whenever the considered time point, the ratio between bioactivation and detoxication pathways is not linear but mainly concentration dependent with a maximum concentration around 10 µM. This confirms the observations of Fig. 7. In addition, although the curve profiles and their associated values are quite similar, subtle variations can be observed. For example, we noticed that the peak shape is more acute at 6 h than at 120 h. Finally, these simulations suggest that the family of 73 ODE models have similar behaviors when the dose of MeIQx is greater than 100 µM, since the variability of the predictions for such dose is very low.

### Simulations of the distribution coefficients dependency to MeIQx suggest that both CYP1A2 and UGT regulation occur at low concentrations of MeIQx

**Figure 9:**
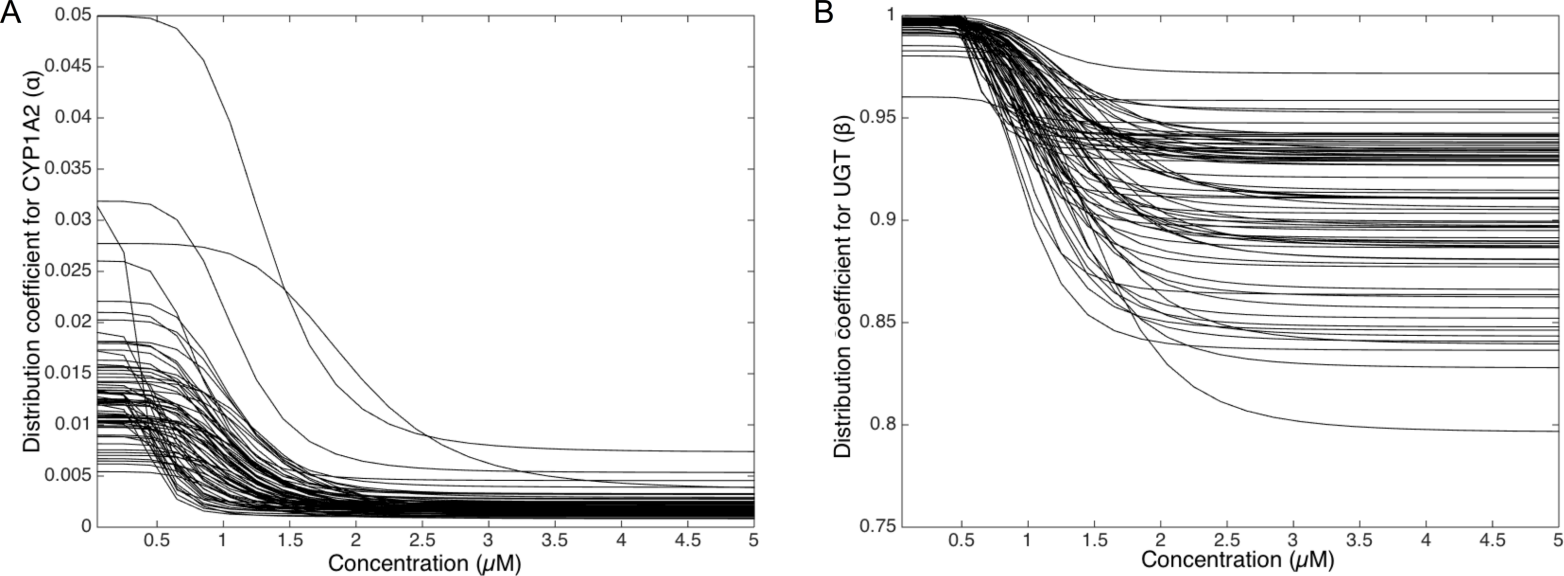
Variation of the CYP1A2 distribution coefficient *α* (A) and the UGT distribution coefficient *β* (B) modeled by a sigmoid function of the MeIQx concentration. All 73 parameterized numerical models which explain the best the transformation of MeIQx into several compounds were built by introducing a MeIQx-dependant distribution coefficient for CYP1A2 and UGT modeled with a sigmoid function. The evolution of this dependency with respect to the concentration of MeIQx is depicted for both enzymes.

To deepen the regulation mechanism, we plotted the variation of the CYP1A2 distribution coefficient *α* (Fig. 9A) and the UGT distribution coefficient *β* (Fig. 9B) with respect to the MeIQx concentration. Let us recall that all the 73 parameterized numerical models which explain the best the transformation of MeIQx into several compounds were built by introducing a MeIQx-dependent distribution coefficient for CYP1A2 and UGT modeled with a sigmoid function. The evolution of this dependency with respect to the concentration of MeIQx is depicted in Fig. 9. Our simulations suggest that the distribution coefficient for CYP1A2 ranges from 0.005 at high concentrations of MeIQx to 0.05 at low concentrations of MeIQx. This suggests that the distribution of CYP1A2 always favors the bioactivation pathway, but that for MeIQx concentrations lower than 0.5 µM, the relative quantity of CYP1A2 involved in detoxification in multiplied by a factor 10. A possible interpretation is that the low quantity of CYP1A2 allows the system to favor detoxification pathway although this pathway is kinetically disadvantaged. This is consistent with experimental measurements of enzymatic activities by Turesky et al. (2001) evidencing that the detoxification pathway kinetics is slower than the bioactivation pathway.

Similarly, our simulations suggest that the UGT distribution coefficient highly favors the direct transformation of MeIQx in glucoronic compounds with respect to the transformation of intermediary compounds HONH-MeIQx into glucuronic compounds. Although, this tendency is leverage at high dose of MeIQx, with an inflection point at 1.25 µM. This suggests that the system needs to transform very toxic intermediary metabolites HONH-MeIQx when their concentration is too high.

Together, the simulation of the distribution coefficients according to MeIQx concentration suggest that the following scenario of regulation holds. For initial dose greater than 2 µM, CYP1A2 is mainly involved in the catalysis of the bioactivation pathway whereas UGT in involved in the transformation of intermediary compounds HONH-MeIQx. When the concentration of MeIQx reaches a threshold on 1.25 µM, UGT is redistributed to favor a direct transformation of MeIQx into glucoronic compounds. Finally, when the concentration of MeIQx reaches 0.75 µM, CYP1A2 favors the catalysis of the most energetic consuming detoxification pathways to gain in efficiency. Although the thresholds (inflection points) are clearly patient dependent, it should be noted that these phenomena occur in a range of MeIQx corresponding to potential real exposure.

### Influence of CYP1A2 on balance between bioactivation and detoxification

**Figure 10:**
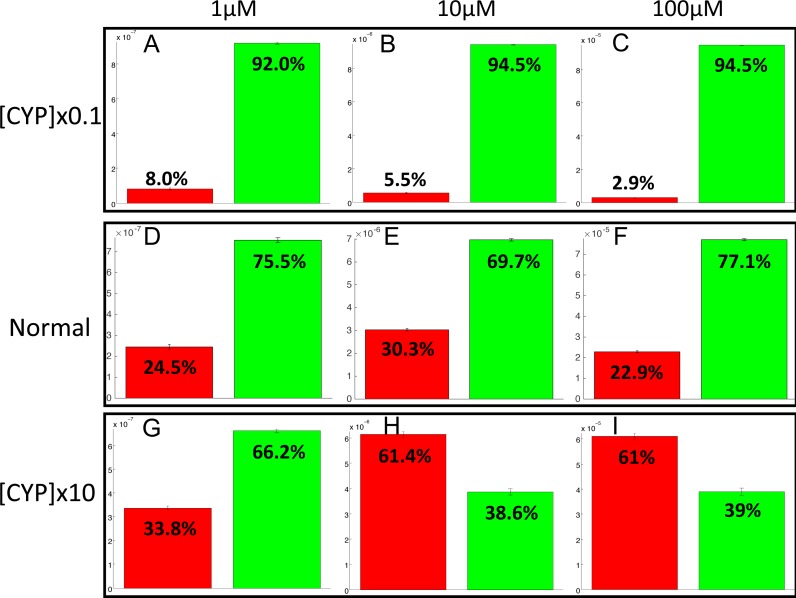
Evaluation of bioactivation and detoxification products formation in function of the total CYP1A2 concentration at 180 h of MeIQx exposure. All 73 models were simulated using three different initial dose of MeIQx (1 (A, D, E), 10 (B, E, H) and 100 (C, F, I) µM) during 180 h of MeIQx exposition. Simulations were performed in three different conditions: a normal condition where the total CYP1A2 concentration of MeIQx corresponds to the parameter values learned for the 73 models (D, E, F), a low-concentration condition where the CYP1A2 concentration of each of the 73 models is decreased by a factor 10 with respect to the initial models (A, B, C), and high-concentration condition where the CYP1A2 concentration of each of the 73 models is increased by a factor 10 (G, H, I). After 180 h, the average values of the bioactivation and detoxification ratio was calculated among the 73 models.

The PotterWheel matlab toolbox was used to perform a 2D sensibility analysis of the influence of each parameter over the system. As depicted in Fig. S1, this analysis highlighted that the total concentration of CYP1A2 is a key actor of bioactivation products formation. In order to illustrate and understand better the influence of this parameter, the parameter value of the total concentration of CYP1A2 was modified in the 73 models (multiplication by 10, and division by 10). The perturbed models were simulated in order to compute the balance between bioactivation and detoxification. The results of these simulations is depicted in Fig. 10. By decreasing the concentration of CYP1A2 by a factor of 10, we observe that that the detoxification pathway largely predominates, so that the bioactivation pathway is minor and accounts for less than 10% of the total metabolism. In addition, the complete MeIQx metabolism is not reached at 180 h for a concentration of 100 µM. On the contrary, by increasing the concentration of CYP1A2 by a factor of 10, we observe that that the bioactivation predominates for high concentration. At low concentration, the detoxification still predominates despite the increasing of the importance of the bioactivation pathway. This suggests that, together with the concentration of MeIQx, CYP1A2 is a major actor in the regulation of the balance between bioactivation and detoxification, and that both its concentration and its operating mode are crucial to modulate the balance.

## Discussion

In order to study the behaviour of the environmental contaminant MeIQx in hepatocytes, we developed a mathematical model to evaluate the balance between bioactivation and detoxification according to MeIQx concentration and time exposures.

The main originality of our modeling approach is that we did not select a single set of parameter values fitting with the experimental data. As discussed in Villaverde & Banga, (2014); Brown & Sethna, (2003); Gutenkunst et al., (2007), the number of time point data sets are insufficient when compared to the number of parameters to precisely estimate them. Therefore, to gain in robustness and limiting bias in the parameter selection process (Krauss et al., 2013), we selected all parameterized models with a *p*-value equal to 1 in the Potterswheel toolbox (Maiwald & Timmer, 2008). This *p*-value is a statistical score implemented in Potterswheel to determine if a model is rejected or not (Maiwald & Timmer, 2008).

We showed that the model (consisting of a family of 73 parameterized ODE) can predict the balance between bioactivation and detoxification of MeIQx in human hepatocytes at a large range of concentrations. Interestingly, the prediction MeIQx are more robust at high concentrations than at low concentrations, where the ratio curves displayed a wider dispersion. The dispersion at low concentration can be explained by the great variability of local fitting scores among all parameterized models. Let us emphasize that such a dispersion is consistent with the results obtained by Gu et al.(2010) and Stillwell et al.(1999) which showed an important variability for the metabolism of C-hydroxy-MeIQx and HON-MeIQx-N^2^-Gl at low concentrations.

A characteristic shared by the 73 sets of parameters that were retained from the model identification process is that the *Km* values ranged between 2.21 and 7 µM for *Km*_*IQx*−8−*COOH*_ (C-Hydroxy-MeIQx) and between 2.45 and 4.12 µM for Km_*HONH*−*MeIQx*_ (N-Hydroxy-MeIQx) (see Fig. S2). The range of values of these parameters (µM) is in accordance with human exposure (Yuan et al., 2002; Iwasaki et al., 2004). In addition, we noticed that these parameter values are close to each other in all parameters sets. This suggests that both bioactivation and detoxification catalyzed by CPY1A2 have a close affinity. We noticed that both reactions consist in a transfer of a hydroxyl-group from the Heme of CYP1A2 to a site of metabolism of the MeIQx molecule. This transfer occurs in the catalytic site of CYP1A2. This suggests that the affinities of bioactivation and detoxification are related to the position of the MeIQx in the catalytic site. A simulation of the 3D molecule conformation with a docking approach using the SwissDock software (Grosdidier, Zoete & Michielin, 2011b; Grosdidier, Zoete & Michielin, 2011a) confirmed that the positions of carbon (C) or azote (N) near the Heme have equivalent energies (see Fig. S3). This may provide a plausible explanation for the similar affinity constant (Km) values.

The evolution of the bioactivation/detoxication ratio displayed a non-monotonous curve: according to the model predictions, MeIQx toxicity differs in favor of bioactivation pathway around 10 µM. The abrupt change in the ratio suggests that regulatory mechanisms of the bioactivation or detoxification pathways might occur. A plausible explanation would be the saturation of the detoxification pathway at first and next the redistribution of CYP1A2 towards the bioactivation pathway. We tested this hypothesis by performing a parameter inference procedure, assuming that the parameters *α* and *β* which depict the distribution of CYP1A2 and UGT were constant. As depicted in Table 2, the best-fit parameters of such models never fitted with biological data. This suggests that the CYP1A2 distribution between the two pathways did not result from saturation effects. Because fitting the model requires a dynamical distribution of coefficients (*α* and *β*) for CYP1A2 and UGT, respectively, complex mechanisms might modulate enzyme activities. A mechanism might be a change of regioselectivity of CYP1A2 for MeIQx. Such a behavior has been previously described for the metabolism of Bisphenol A in rat hepatocytes where UGT followed a biphasic evolution involving two distinct *Vm* values (Pritchett, Kuester & Sipes, 2002). According to this observation, introducing a sigmoid function in the Michaelis Menten equation appears to be a suitable approach to model the CYP1A2 and UGT dynamics and their dependency on the substrate concentration.

In order to confirm the role of CYP1A2 in the balance between bioactivation and detoxification, we performed several simulations based on CYP1A2 perturbations. Our results suggest that modifying only the total concentration of CYP1A2 in the model yield a complete distortion of the balance between bioactivation and detoxification. Highly decreasing the total CYP1A2 concentration yields an important reduction of the bioactivation. On the contrary, increasing the total concentration of CYP12 impacts the balance in favor of bioactivation, although this effect is moderate at low concentrations of MeIQx. This can be explained by the choice of sigmoid curves, which favor the detoxification pathway by C-Hydroxy-MeIQx at low concentrations (around 1 µM).

To conclude, our results demonstrated that the detoxification pathway predominates for a wide concentration range (from 0.05 to 100 µM) reinforcing the evidence of liver detoxification function. Depending of the concentrations of MeIQx, the ratio between bioactivation and detoxification pathways is highly modulated. This ratio depicts the balance between the quantity of MeIQx metabolized either in detoxification products or transformed in Potential-Genotoxic-Compound. Obviously, our model provides an over-approximation of the risk of bioactivation since the model assumes that the system is closed and that all non-measured compounds are potentially genotoxic metabolites and therefore may bind to DNA adducts. However, our analysis suggests that the predicted ratio between bioactivation and detoxification pathways could be considered as an indicator of MeIQx genotoxicity at low concentrations of MeIQx. More importantly, our model clearly illustrates the importance of introducing regioselectivity phenomena in in-silico modelling approaches. Indeed, our analyzes suggest that this mechanism may occur on CYP1A2 regulation and therefore highly impact the balance between bioactivation and detoxification.

Future work will mainly consist of testing the relevance of our modelling approach in a wider context of multi-organ global exposition. Indeed, a main limitation of the model is that is restrained to human hepatocytes since the parameters were learned according to hepatic metabolism data. Although the liver is known to be the major organ of detoxification (justifying the choice of developing a model for this organ) and our predictions are by extrapolation consistent with data coming from oral exposition, it will be highly interesting to compare the MeIQx dynamics in other human organs as soon as the corresponding datasets are available. Another obvious limitation of the model is that it is patient dependent since the fitting was based on datasets extracted from human patients with no information on patient characteristics such as enzyme polymorphism. In this setting, the available data are not sufficient to take bioavailability and tissue distribution into account and to implement a population model. Nonetheless, we consider that our approach is a first seminal step to pave the way towards the development of complex models (Zhuang & Lu, 2016; Kuepfer et al., 2016) by pointing out the importance of integrating complex models for enzyme regulation in multi-organ PBPK models.

##  Supplemental Information

10.7717/peerj.3703/supp-1 Table S1 Equivalence of Gu’s data in molar concentration We compared our predicted data with those of Gu et al. (2010). They measured the remaining MeIQx, 8-CH2OH-IQx and IQx-8-COOH in human urine collected at 10 h after consumption of cooked beef. To compare our predicted data of C-hydroxy-MeIQx pathway with Gu’s data, we add up the 8-CH2OH-IQx and IQx-8-COOH of Gu’s data and use the following relation linking mass (m) with molar concentration (C), molecular weight (M) and volume (V) : *m* = *C* × *M* × *V*. As references, we use a molecular weight for MeIQx of 213.23854 g/mol extracted from PubChem Compound record, CID: 62275 (for Biotechnology Information. PubChem Compound Database, ties), 920 ng as the average amount (m) of MeIQx ingested in Gu’s study, a volume of 10 mL corresponding of the medium volume in Langouet’s experiments, and a concentration of 50 µM.

10.7717/peerj.3703/supp-2 Table S2 Search space of the different parameters The ODE model needs a search space for each parameter of the model in order to run fitting algorithm. The boundary for each parameter is specified in this present table.

10.7717/peerj.3703/supp-3 Figure S1 2D sensitivity analysis performed by PottersWheel toolbox All parameters have been 693 disturb by a factor 10 of a 2D sensitive analysis in order to study the bioactivation Response by the formation of Potential-Genotoxic-Compound. The illustration illustrates only the most sensitive parameters and the UGT case by comparison.

10.7717/peerj.3703/supp-4 Figure S2 Variation of the searched parameters for the 73 fit sequences selected after filtering Note that n1 and n2 for the sigmoid function associated with the distribution of CYP1A2 and UGT are fixed to 5. The Kms, and concentrations are expressed in Molar, k in h^−1^ and Vms in M h^−1^

10.7717/peerj.3703/supp-5 Figure S3 Docking of the MeIQx with the CYP1A2 by using SwissDock The first conformation with the CH3 group near the Heme with an energy −4.53653 kcal/mol (A) and the second conformation with the NH2 group near the Heme with an energy of −3.93616 kcal mol (B). Resulting in these data, the two conformations are closed but the conformation with the CH3 group near the heme seems to be privileged.

## Additional Information And Declarations

### Data Availability

The following information was supplied regarding data availability: The raw data are included in Figs. 2, 3 and Table S2.
