# Supplementary material for: A modeling approach to evaluate the balance between bioactivation and detoxification of MeIQx in human hepatocytes
Source: PeerJ. 2017 Sep 1;5:e3703. doi: 10.7717/peerj.3703 (PMC5582613; doi:10.7717/peerj.3703)
Supplement: Table S1 — We compared our predicted data with those of Gu et al. (2010). They measured the remaining MeIQx, 8-CH2OH-IQx and IQx-8-COOH in human urine collected at 10 h after consumption of cooked beef. To compare our predicted data of C-hydroxy-MeIQx pathway with Gu’s data, we add up the 8-CH2OH-IQx and IQx-8-COOH of Gu’s data and use the following relation linking mass (m) with molar concentration (C), molecular weight (M) and volume (V) : m = C × M × V. As references, we use a molecular weight for MeIQx of 213.23854 g/mol extracted from PubChem Compound record, CID: 62275 (for Biotechnology Information. PubChem Compound Database, ties), 920 ng as the average amount (m) of MeIQx ingested in Gu’s study, a volume of 10 mL corresponding of the medium volume in Langouet’s experiments, and a concentration of 50 µM. [file peerj-05-3703-s001.pdf]

	Equivalence relation		Subject									
		Total	S-001	S-003	S-005	S-008	S-009	S-012	S-013	S-014	S-015	S-020
% Dose		100	56	64.8	68.7	59.4	54.1	66.2	65.4	35.8	71.9	73.1
Mass (μg)	106.62	0.92	0.5152	0.5962	0.6320	0.5465	0.4977	0.6090	0.6017	0.3294	0.6615	0.6725
Molar concentration (μM)	50	0.4314	0.2416	0.2796	0.2964	0.2563	0.2334	0.2856	0.2822	0.1545	0.3102	0.3154
