# Supplementary material for: A modeling approach to evaluate the balance between bioactivation and detoxification of MeIQx in human hepatocytes
Source: PeerJ. 2017 Sep 1;5:e3703. doi: 10.7717/peerj.3703 (PMC5582613; doi:10.7717/peerj.3703)
Supplement: Table S2 — The ODE model needs a search space for each parameter of the model in order to run fitting algorithm. The boundary for each parameter is specified in this present table. [file peerj-05-3703-s002.pdf]

Parameter name	initial value	type	minValue	maxValue
k1	0.1	global	1e-01	10000
k2	0.1	global	1e-01	10000
k3	0.1	global	1e-15	10000
k4	0.1	global	1e-15	10000
$Vm_{[MeIQx-N^2-SO3H]}$	0.1	global	1e-15	1e-6
$Vm_{oxo-MeIQx}$	0.1	global	1e-15	1e-6
$Vm_{Potential-Genotoxic-Compound}$	0.1	global	1e-15	1e-6
$Km_{IQx-8-COOH}$	0.1	global	1e-07	0.0001
$Km_{HONH-MeIQx}$	0.1	global	1e-07	0.0001
$Km_{MeIQx-N^2-Gl}$	0.1	global	1e-7	1e-3
$Km_{[HONH-MeIQx-N^2-Gl]}$	0.1	global	1e-7	1e-3
$Km_{[MeIQx-N^2-SO3H]}$	0.1	global	1e-7	1e-3
$Km_{oxo-MeIQx}$	0.1	global	1e-7	1e-3
$Km_{Potential-Genotoxic-Compound}$	0.1	global	1e-7	1e-3
CYP	0.1	global	1e-18	1e-8
UGT	0.1	global	1e-18	1e-8
a1	0.1,	global	0	1
b1	0.1,	global	0	1
$\theta1$	0.1	global	1e-9	1e-3
n1	5	fix	5	5
a2	0.1	global	0	1
b2	0.1	global	0	1
$\theta2$	0.1	global	1e-9	1e-3
n2	5	fix	5	5
