# Supplementary figures and images for: A modeling approach to evaluate the balance between bioactivation and detoxification of MeIQx in human hepatocytes
Source: PeerJ. 2017 Sep 1;5:e3703. doi: 10.7717/peerj.3703 (PMC5582613; doi:10.7717/peerj.3703)

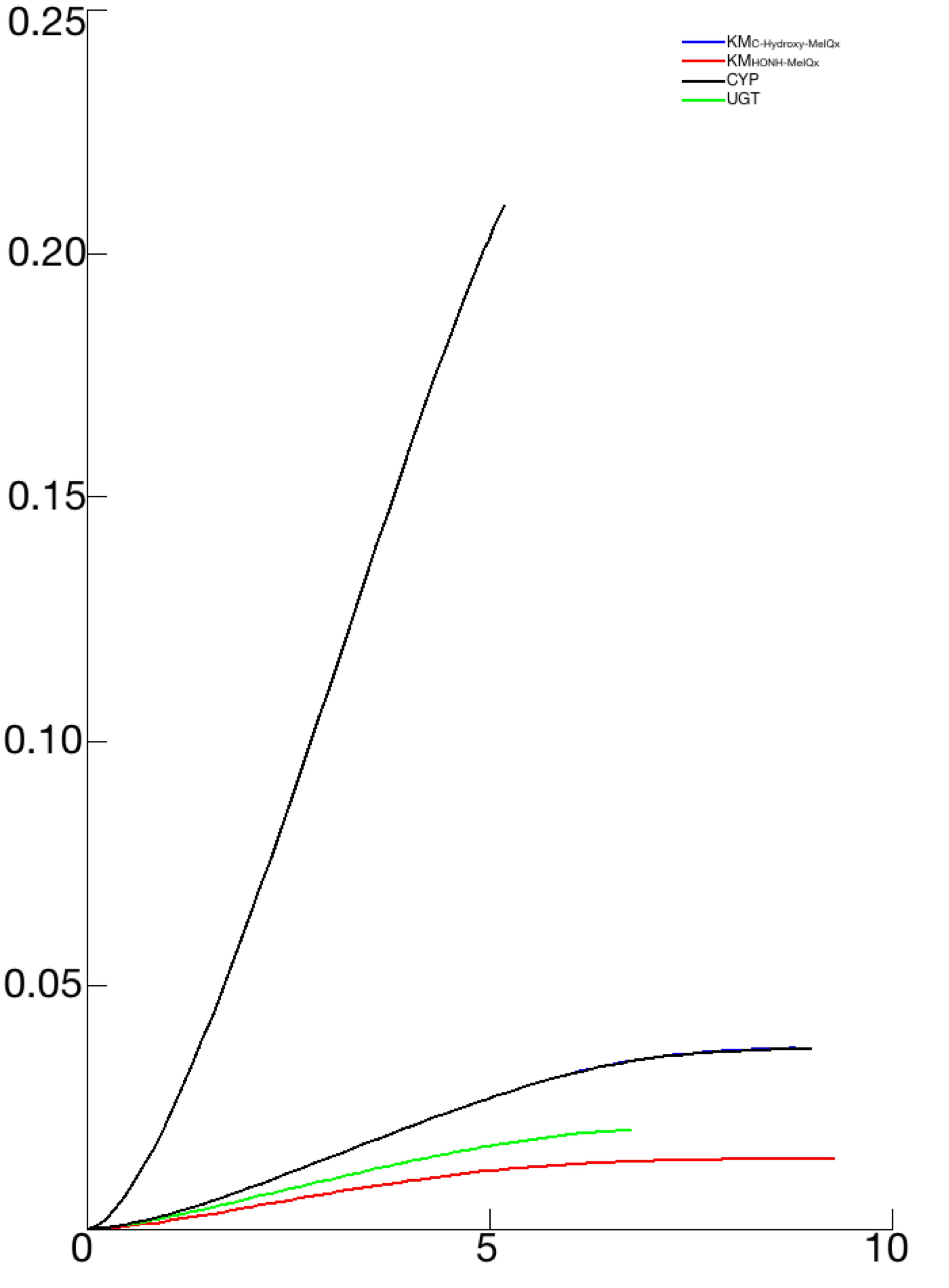

Supplement: Figure S1 — All parameters have been 693 disturb by a factor 10 of a 2D sensitive analysis in order to study the bioactivation Response by the formation of Potential-Genotoxic-Compound. The illustration illustrates only the most sensitive parameters and the UGT case by comparison. [file peerj-05-3703-s003.png]

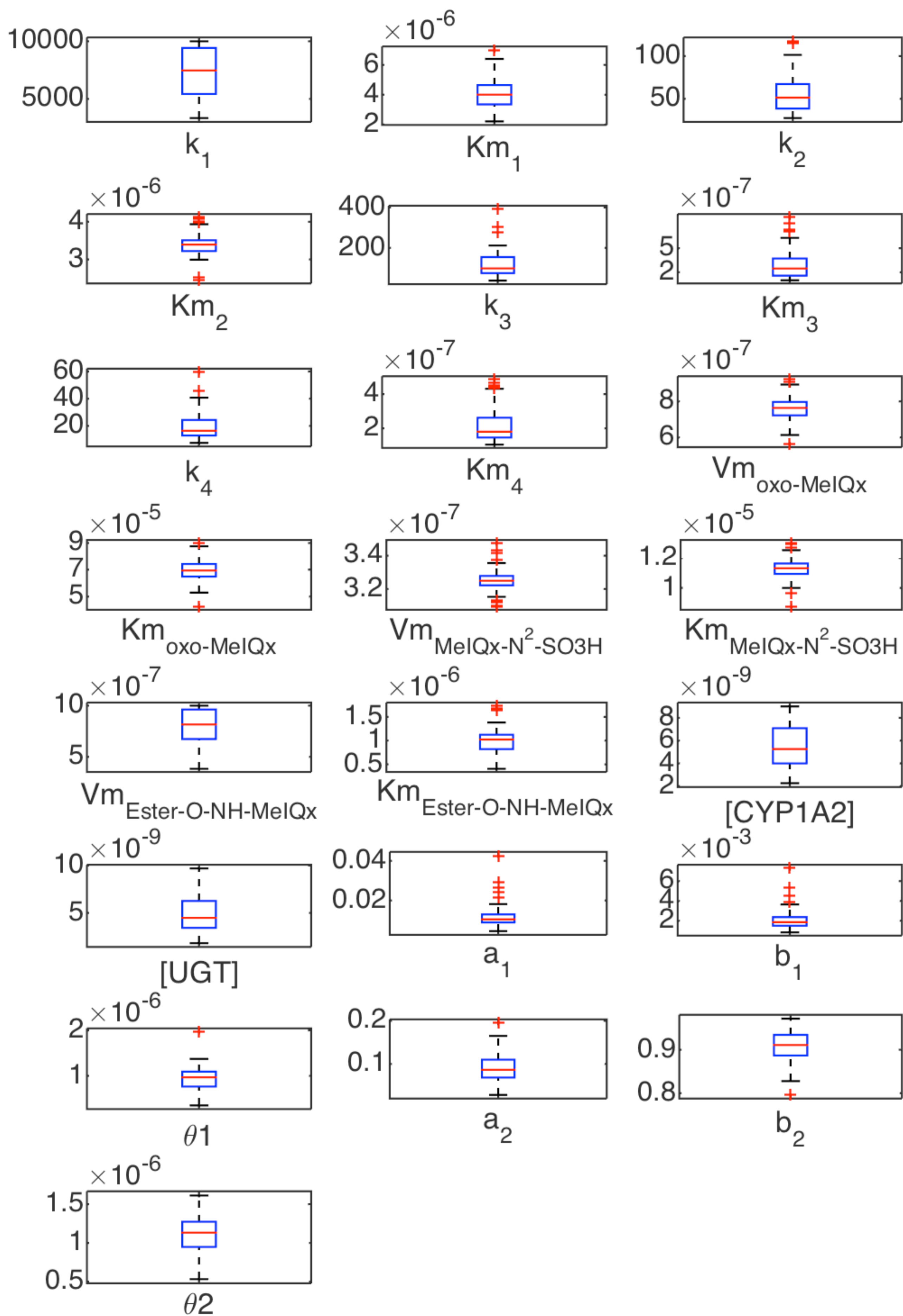

Supplement: Figure S2 — Note that n1 and n2 for the sigmoid function associated with the distribution of CYP1A2 and UGT are fixed to 5. The Kms, and concentrations are expressed in Molar, k in h−1 and Vms in M h−1 [file peerj-05-3703-s004.pdf]
